# Comprehensive analysis of stearoyl-coenzyme A desaturase in prostate adenocarcinoma: insights into gene expression, immune microenvironment and tumor progression

**DOI:** 10.3389/fimmu.2024.1460915

**Published:** 2024-09-16

**Authors:** Jie Wang, Liang Ying, He Xiong, Duan-Rui Zhou, Yi-Xuan Wang, Hui-Lian Che, Zhang-Feng Zhong, Guo-Sheng Wu, Yun-Jun Ge

**Affiliations:** ^1^ MOE Medical Basic Research Innovation Center for Gut Microbiota and Chronic Diseases, Wuxi School of Medicine, Jiangnan University, Wuxi, China; ^2^ Macao Centre for Research and Development in Chinese Medicine, State Key Laboratory of Quality Research in Chinese Medicine, Institute of Chinese Medical Sciences, University of Macau, Macao, Macao SAR, China

**Keywords:** stearoyl-coenzyme A desaturase, prostate adenocarcinoma, gene expression, immune microenvironment, functional enrichment analysis, tumor progression

## Abstract

Prostate adenocarcinoma (PRAD) is a prevalent global malignancy which depends more on lipid metabolism for tumor progression compared to other cancer types. Although Stearoyl-coenzyme A desaturase (SCD) is documented to regulate lipid metabolism in multiple cancers, landscape analysis of its implications in PRAD are still missing at present. Here, we conducted an analysis of diverse cancer datasets revealing elevated *SCD* expression in the PRAD cohort at both mRNA and protein levels. Interestingly, the elevated expression was associated with *SCD* promoter hypermethylation and genetic alterations, notably the L134V mutation. Integration of comprehensive tumor immunological and genomic data revealed a robust positive correlation between *SCD* expression levels and the abundance of CD8^+^ T cells and macrophages. Further analyses identified significant associations between *SCD* expression and various immune markers in tumor microenvironment. Single-cell transcriptomic profiling unveiled differential *SCD* expression patterns across distinct cell types within the prostate tumor microenvironment. The Gene Ontology and Kyoto Encyclopedia of Genes and Genome analyses showed that *SCD* enriched pathways were primarily related to lipid biosynthesis, cholesterol biosynthesis, endoplasmic reticulum membrane functions, and various metabolic pathways. Gene Set Enrichment Analysis highlighted the involvement of elevated *SCD* expression in crucial cellular processes, including the cell cycle and biosynthesis of cofactors pathways. In functional studies, *SCD* overexpression promoted the proliferation, metastasis and invasion of prostate cancer cells, whereas downregulation inhibits these processes. This study provides comprehensive insights into the multifaceted roles of SCD in PRAD pathogenesis, underscoring its potential as both a therapeutic target and prognostic biomarker.

## 1 Introduction

Prostate adenocarcinoma (PRAD) is the second most common cancer among men globally, and the fifth leading cause of cancer-related mortality (1). In China, the situation is also concerning with the rising incidence of prostate cancer (2). Although there have been advancements in surgical techniques, radiotherapy, and newer therapies such as focal ultrasound ablation, androgen deprivation therapy (ADT) remains the cornerstone of standard prostate cancer (PRAD) treatment (3). As aging progresses, PRAD can develop rapidly and advanced PRAD poses significant risks of metastasis, drug resistance, and transformation into castration-resistant prostate cancer. This underscores the paramount importance of timely intervention (4).

PRAD exhibits a unique ‘heterogeneity’ in its lipid metabolism. This characteristic is twofold: firstly, PRAD relies more on lipid oxidation as its primary energy source compared to other tumors, accompanied by a relatively low rate of glucose uptake (5). Secondly, as tumors progress malignantly, there is a notable increase in the activity of fatty acid (FA) synthesis in PRAD cells, highlighted by the elevated expression of key enzymes involved in fatty acid synthesis (6). Epidemiological studies have revealed that conditions such as obesity, metabolic syndrome, diabetes, and systemic metabolic disorders, as well as a high-fat diet, are associated with an elevated risk of PRAD and a decreased survival rate among PRAD patients (7). Consequently, targeting the key processes and enzymes involved in lipid metabolism emerges as a potential strategy for the treatment of malignant PRAD.

Stearoyl-coenzyme A desaturase (SCD, also known as SCD1 or FADS5) is a key rate-limiting enzyme localized in the endoplasmic reticulum (ER) membrane. It catalyzes the desaturation of fatty acids and plays a crucial role in the *de novo* lipogenesis (8). Notably, SCD facilitates the conversion of saturated fatty acids (SFA) to monounsaturated fatty acids (MUFA). Specifically, it converts palmitic acid (PA, C16:0) into palmitoleic acid (POA, C16:1), and stearic acid (SA, C18:0) into oleic acid (OA, C18:1) (9). The unsaturated lipids serve as lipid pools for β-oxidation and as components of the cell membrane, which are indispensable for tumor progression (10). This is demonstrated by the significant overexpression of SCD in various types of cancer (11–14). Therefore, inhibiting SCD functions has been proposed as a therapeutic strategy for some types of cancer (15–18). Despite reports suggesting SCD as a therapeutic target, a comprehensive understanding of its implications in PRAD has not yet been realized.

The tumor microenvironment (TME) consists of immune cells, stromal cells, and the extracellular matrix. Within this complex network, two categories of immune cells emerge as pivotal players: tumor-infiltrating lymphocytes (TILs) and macrophages. Among TILs, CD8+ T cells are central to orchestrating anti-tumor immunity by recognizing and eliminating tumor cells presenting tumor-associated antigens. However, their cytotoxic function is often suppressed by tumor-induced immune checkpoints, such as programmed death-1 (PD-1) and cytotoxic T-lymphocyte-associated protein 4 (CTLA-4) (19–22). Macrophages, another crucial component of the TME, exhibit plasticity by differentiating into either M1 (anti-tumorigenic) or M2 (tumor-promoting) phenotypes. M1 macrophages possess strong phagocytic and immunostimulatory properties that contribute to anti-tumor immunity, while M2 macrophages create an inflammatory and immunosuppressive environment that promotes tumor progression (23, 24). Therapeutic strategies that target immune checkpoints or modulate macrophage polarization to enhance anti-tumor immune responses can improve outcomes for cancer patients.

In this study, we employed bioinformatics approaches to analyze public datasets encompassing clinicopathological data, promoter methylation levels, genetic alterations, immune infiltrations, single-cell transcriptomic data, and pathway enrichment analysis. Additionally, we conducted functional experiments to validate our computational findings. Our findings demonstrated that *SCD* overexpression is correlated with PRAD progression, as well as the changes in the tumor immune microenvironment.

## 2 Materials and methods

### 2.1 UALCAN

The University of Alabama at Birmingham cancer data analysis portal (UALCAN, http://ualcan.path.uab.edu/) is a comprehensive web portal for analyzing cancer omics data (25). In this study, it was used to analyze the difference of *SCD* expression between tumor tissue and normal tissue, and the expression of *SCD* in PRAD was analyzed based on patients’ gender, age, race, tumor protein P53 (TP53) mutation status, lymph node metastasis status, or a molecular signature. The expression level of *SCD* was normalized as transcript per million reads. *P* < 0.05 was considered statistically significant. The abbreviations for the cancers were illustrated in **Supplementary Table S1**.

### 2.2 GEPIA

The Gene Expression Profiling Interactive Analysis (GEPIA) database (http://gepia.cancer-pku.cn/) consists of RNA sequencing expression data of 9,736 tumors and 8,587 normal samples derived from The Cancer Genome Atlas (TCGA) and the Genotype-Tissue Expression (GTEx) databases (26). It was used to analyze the differential mRNA expression of *SCD* between PRAD, colon adenocarcinoma (COAD), liver hepatocellular carcinoma (LIHC), or uterine corpus endometrial carcinoma (UCEC) tissues and their corresponding normal tissues.

### 2.3 The human protein atlas

The Human Protein Atlas (HPA, https://www.proteinatlas.org/) provides comprehensive data on human proteins in cells and tissues using various omics techniques (27). The database consists of 26,941 antibody proteome data for 17,165 unique proteins. PRAD and adjacent tissue sections were immunostained with SCD antibodies (Sigma-Aldrich, HPA012107, Rabbit).

### 2.4 Immunohistochemistry

The tissue microarray (TMA) containing PRAD, related urological tumors, and adjacent normal tissues was purchased from Outdo Biotech (Shanghai, China). The TMA (HUrSC060PT01) contained 60 tissue samples, including 36 tumor samples. SCD expression in the patient-derived normal and tumor samples was analyzed by immunohistochemistry (IHC). The IHC staining was performed using a SCD-specific antibody included in the IHCeasy SCD Ready-To-Use IHC kit (Proteintech; Wuhan, China). The experiments were carried out according to the manufacturer’s instructions. The IHC images were captured with the Pannoramic MIDI II Digital Slide Scanners (3DHISTECH Ltd., Budapest, Hungary). H-score was used to evaluate the IHC results, the principle of which was to use ImageJ software (Rawak Software Inc, Stuttgart, Germany) to analyze automatically the intensity and proportion of immunostaining. Grading scale was defined in a semiquantitative manner, as follows: 0, Negative; 1+, Low Positive; 2+, Positive; and 3+, High Positive. The formula was used to calculate H-score: H-score = (percentage of 1+ cells × 1) + (percentage of 2+ cells × 2) + (percentage of 3+ cells × 3).

### 2.5 cBioPortal

The cBio Cancer Genomics Portal (cBioPortal, http://www.cbioportal.org/) provides data for more than 5,000 tumor samples from 20 cancer studies (28). The alteration frequency of SCD mutations in the genomic profiles was used to calculate Pearson’s correlation coefficients across pan-cancer, along with the data on missense mutation sites in PRAD.

### 2.6 CancerSEA

CancerSEA (http://biocc.hrbmu.edu.cn/CancerSEA/) was created to decode Pearson correlations between 14 different functional states and relevant genes in human malignancies, containing gene sequencing profiles of 41,900 cancer single cells (29). In this study, we used CancerSEA to investigate the relationships between the *SCD* genes and the afore mentioned PRAD functional states at the single-cell level.

### 2.7 TIMER

Tumor immune estimation resource (TIMER, https://cistrome.shinyapps.io/timer/) is a database for comprehensive analysis of immune infiltrates across diverse cancer types (30), which consists of 10,897 samples from 32 cancer types. It enables the evaluation of the correlation between the transcriptional level of *SCD* and the abundances of six immune infiltrates (B cells, CD4^+^ T cells, CD8^+^ T cells, neutrophils, macrophages and dendritic cells), as well as correlations between transcriptional level of *SCD* and immune cell markers. The gene expression level was assessed using log_2_ TPM.

### 2.8 GeneMANIA

The GeneMANIA database (http://www.genemania.org/) is a web-based tool for generating hypotheses about gene function, analyzing gene lists, and prioritizing genes for functional testing (31). It was used to explore the 20 genes that are associated with *SCD*, which have physical interactions, co-expression, predicted gene-gene relationships, pathway relationships, co-localization, genetic interactions, and shared protein domains.

### 2.9 TISCH

Tumor immune single-cell hub (TISCH, http://tisch.comp-genomics.org/) is a single-cell RNA sequencing database focusing on the tumor microenvironment (TME) (32), which provides detailed cell-type annotation at the single-cell level, consisting of 6,297,320 cells and 190 datasets. It was used to analyze *SCD* expression in PRAD datasets, including PRAD_GSE137829, PRAD_GSE141445, PRAD_GSE150692 and PRAD_GSE172301.

### 2.10 Related-gene enrichment analysis

The Gene Ontology (GO, http://www.geneontology.org/) database and Kyoto Encyclopedia of Genes and Genome (KEGG, http://www.genome.jp/kegg/) database were employed for the enrichment analysis of *SCD* and its 20 related genes. They were performed with the Database for Annotation, Visualization and Integrated Discovery (DAVID, https://david.ncifcrf.gov/) (33), focusing on the key phenotypes and signal pathways.

### 2.11 GSEA analysis

The Gene Set Enrichment Analysis (GSEA) analysis was implemented to further investigate the potential mechanisms of *SCD* behind the initiation and progression of PRAD (34). Samples in the TCGA-PRAD dataset were classified into two groups (high-expression group and low-expression group) based on the average expression of *SCD*. A false discovery rate (FDR) < 0.05 and P < 0.05 were considered as criteria for significantly enriched gene sets.

### 2.12 Cell culture

Two PRAD cell lines, PC3 and LNCaP cells, were obtained from the National Collection of Authenticated Cell Cultures (Shanghai, China). Both of the cells were cultured in 1640 (Sigma, St. Louis, MO, USA) with 10% fetal bovine serum (HyClone, Logan, Utah, USA), 100 U/ml penicillin and 100 mg/ml streptomycin. Cells were maintained at 37°C in a humidified environment in an incubator with 5% CO2.

### 2.13 Small interfering RNA

Small interfering (si) RNAs used in this study were purchased from Geenchem (Shanghai, China). The siRNA sequences were as follows: negative control (NC) (Forward: 5’-UUCUCCGAACGUGUCACGUdTdT-3’; Reverse: 5’-ACGUGACACGUUCGGAGAAdTdT-3’), and si-*SCD* (Forward: 5’-GCACAUCAACUUCACCACATT-3’; Reverse: 5’-UGUGGUGAAGUUGAUGUGCTT-3’). Lipofectamine 8000 from Beyotime (Shanghai, China) was used to transfect siRNA into PC3 and LNCaP cells according to the manufacturer’s instructions.

### 2.14 Lentiviral overexpression

Lentivirus transduction was applied to establish *SCD* overexpression cell lines. *SCD*-inserted lentiviral vector was obtained from Hanbio Biotech (Shanghai, China), and *SCD* was transduced into PC3 and LNCaP cells according to the manufacturer’s instructions. In short, the cells were seeded in a 12-well plate, incubated overnight, and then infected for 24 hours. The *SCD*-overexpression vector carries the green fluorescent protein (GFP) reporter gene, flag-tagged *SCD* gene and the puromycin resistance gene. Transduction efficiency was analyzed with a fluorescence microscope. SCD overexpression in PC3 and LNCaP cell lines was confirmed by Western blot with a SCD-specific antibody.

### 2.15 Western blot

PC3 and LNCaP cells with siRNA or lentivirus transfection were lysed with a cell lysis buffer containing protease inhibitors (Beyotime, Shanghai, China). Protein concentrations were determined by using the BCA Protein Assay Kit (BIO - RAD, California, USA). Protein samples were loaded for electrophoresis on SDS–PAGE and transferred to PVDF membranes (BIO - RAD, California, USA). After blocking with 5% non - fat milk, the membranes were treated with primary antibodies (1:1000 dilution) and then secondary antibodies (1:3000 dilution). All antibodies were purchased from Cell Signaling Technology (Beverly, MA, USA). Finally, protein membrane was treated with an enhanced chemiluminescence detection buffer, and protein bands were captured with the ChemiDOC MP (BIO - RAD, California, USA).

### 2.16 Cell viability

Cell proliferation was analyzed by 3 - (4,5 - dimetrylthiazol) - 2,5 - diphenyltetrazolium bromide (MTT) assay. Exponentially growing cells with siRNA or lentivirus transfection were seeded into 96 - well plates. MF438 was purchased from MedChem Express (New Jersey, USA). MTT was added to each well and the plates were incubated for an additional 4h. The formazan crystal was dissolved by adding 100 μl DMSO. The light absorbance value was measured at 490 nm using a BioTek Synergy H4 all-in-one microplate reader (Vermont, USA).

### 2.17 Migration assay

PC3 and LNCaP cells with siRNA or lentivirus transfection were inoculated in a 6-well plate and cultured overnight. When the cells fused to a monolayer, a sterile suction head with the same width was used to make a “+” scratch. The cells were washed with PBS and incubated for certain time periods. The plate wells were captured with a microscope and the scratch widths were analyzed with the ImageJ software at different time points.

### 2.18 Invasion assay

24-well Transwell chambers with 8-µm membrane filters (Corning Incorporated) were precoated with Matrigel (Yeasen Biotech Co, Ltd, Shanghai, China). The cells were seeded into the upper chamber in serum-free medium, whereas the lower chamber was filled with complete medium containing 10% FBS. Following incubation for 24 h at 37˚C, cells on the lower surface were fixed with 4% paraformaldehyde for 10 min and stained with 0.1% crystal violet for 10 min at room temperature. The stained cells were photographed and quantified.

### 2.19 Statistical analysis

All *in vitro* experiments were repeated at least three times, and the results were presented as the mean ± standard deviation (SD). Student’s *t*-test was used to analyze the significance of the differences. Data were analyzed with GraphPad Prism 7.0 (GraphPad Software, San Diego, CA, USA). *P* < 0.05 was considered statistically significant.

## 3 Results

### 3.1 Pan-cancer analysis of *SCD* expression at the mRNA and protein level

**Figure 1:**
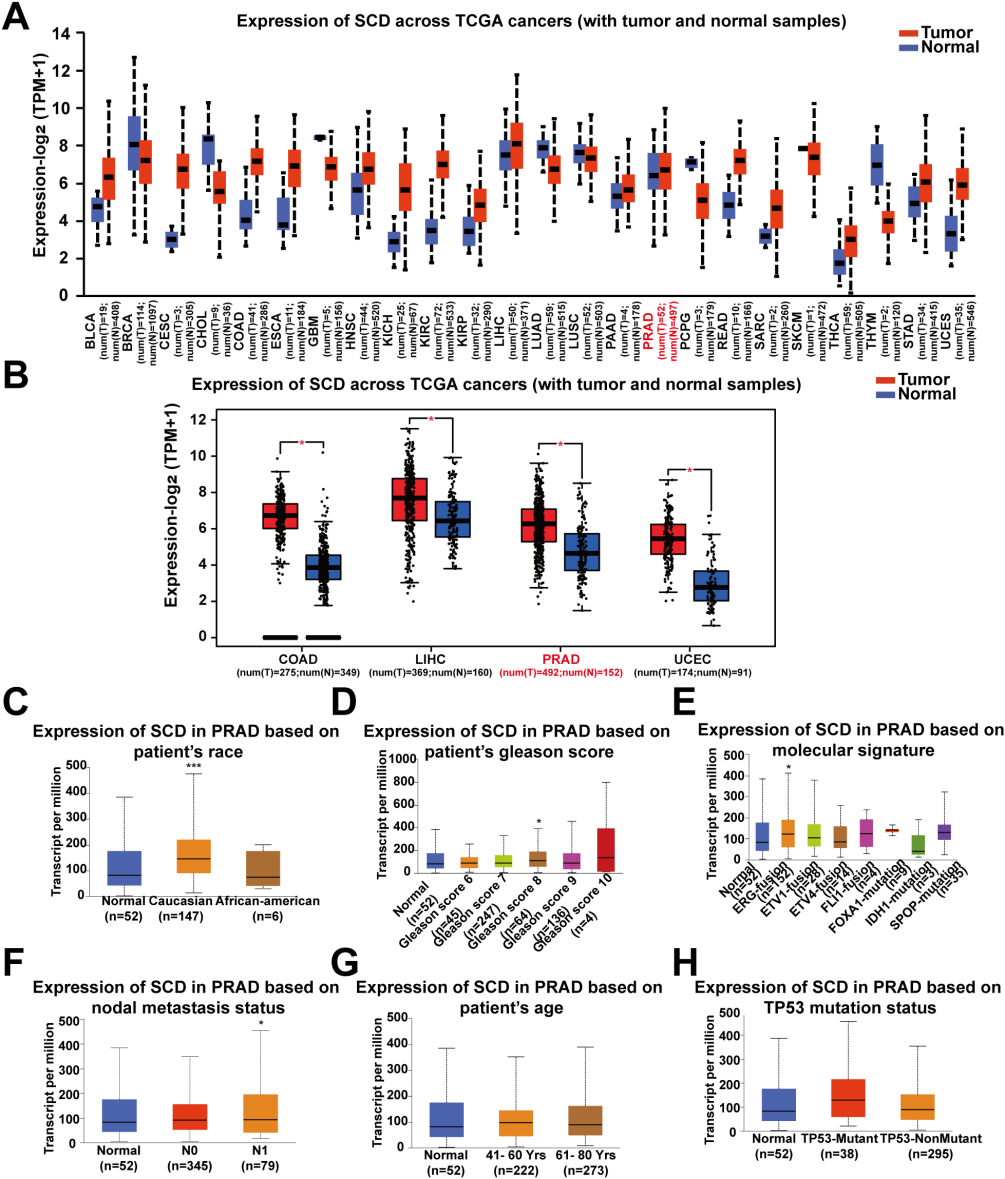
Pan-cancer analyses of the upregulated *SCD* expression at the mRNA level. **(A)** *SCD* expression in the UALCAN database. The mRNA expression between tumors and adjacent normal tissues in 24 types of cancers was analyzed. **(B)** *SCD* expression in the GEPIA database. The mRNA expression between COAD, LIHC, PRAD and UCEC cancers and normal tissues was analyzed and matched TCGA normal and GTEx data. Four-way analysis of variance (ANOVA), *P*< 0.01 and |log_2_FC| > 1 are considered differentially expressed genes. *SCD* mRNA expression in PRAD subtypes with different clinicopathological characteristics, including patients’ race **(C)**, Gleason score **(D)**, molecular signature **(E)**, nodal metastasis status **(F)**, age **(G)** and *TP53* mutation status **(H)** using the UALCAN database. Multiple groups were compared using one-way ANOVA followed by Bonferroni’s multiple comparisons for every two groups. *P <0.05; ***P <0.001.

Pan-cancer analysis of *SCD* transcriptional expression was conducted using the UALCAN platform based on TCGA data. It was revealed that *SCD* expression was upregulated in 24 types of tumors compared to adjacent normal tissues, including PRAD and several other cancer types (**Figure 1A**). Re-examination using an alternative online tool, GEPIA, confirmed these findings, indicating an upregulation of *SCD* expression in PRAD and other cancers (**Figure 1B**). Compared to other tumors, PRAD relies more heavily on lipid oxidation as its primary energy source. Increasing evidence suggests that the lipid synthesis pathway in PRAD is highly activated with the upregulated expression of *SCD*. Therefore, we focused on PRAD to analyze the role of SCD in cancer pathogenesis.

We next determined the correlation of *SCD* transcriptional expression levels with various clinicopathological characteristics in PRAD samples using UALCAN analysis. The results demonstrated elevated *SCD* expression in specific subgroups of PRAD patients, including those of Caucasian race (**Figure 1C**), those with a Gleason score of 8 (**Figure 1D**), those displaying the ERG-fusion molecular signature (**Figure 1E**), and those with N1 nodal metastasis status (**Figure 1F**). In terms of patient’s age and *TP53* mutation status, there was also a trend indicating that higher *SCD* expression was associated with older age (**Figure 1G**) and *TP53* mutation (**Figure 1H**). These findings suggest that *SCD* holds potential as a diagnostic tumor marker for PRAD and highlight the importance of considering different clinical parameters for a comprehensive understanding of its role in PRAD.

**Figure 2:**
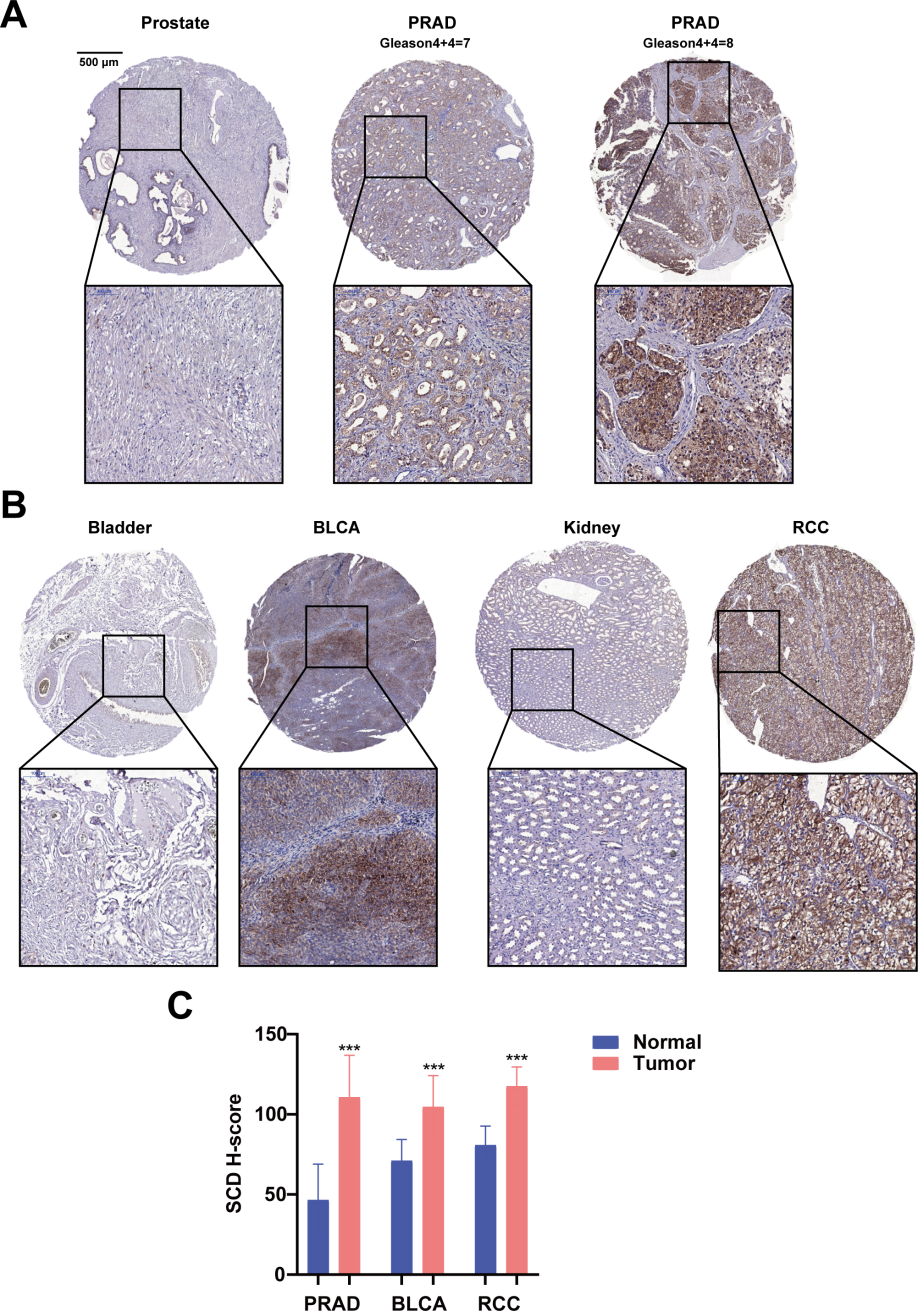
Immunohistochemical analyses of SCD protein expression in PRAD and related urological cancers. **(A)** SCD expression in normal prostate and PRAD. Gleason scores of PRAD were indicated. **(B)** SCD expression in related urological cancers. Bladder urothelial carcinoma (BLCA) and Renal cell carcinoma (RCC) were compared with the respective adjacent normal tissues. **(C)** Quantification of SCD IHC staining intensities. The protein levels of SCD were analyzed with the IHC intensities based on the histoscore algorithm (H-score). ***P < 0.001.

Furthermore, the translational expression level of SCD in pan-cancer was investigated on the HPA platform. The protein expression level of SCD in colorectal cancer, breast cancer, prostate cancer was higher than those in the adjacent normal tissues (**Supplementary Figures S1A, B**). Quantification of the online data revealed that over 50% of cancer cases exhibited medium to high expression levels of SCD (**Supplementary Figure S1C**). To validate the online data, SCD expression in PRAD and related urological tumors was analyzed using a tissue microarray (TMA) by IHC. In patient-derived tissues, SCD expression was upregulated in many urological cancers, including PRAD and renal cancer, compared to adjacent normal tissues (**Figures 2A, B**; **Supplementary Figure S2**). It is worth noting that SCD expression increased as the Gleason scores of PRAD increased (**Figure 2A**). Quantification of our IHC data confirmed that the protein expression level of SCD was upregulated in PRAD (**Figure 2C**).

### 3.2 DNA methylation and genetic alterations related with clinicopathological characteristics

**Figure 3:**
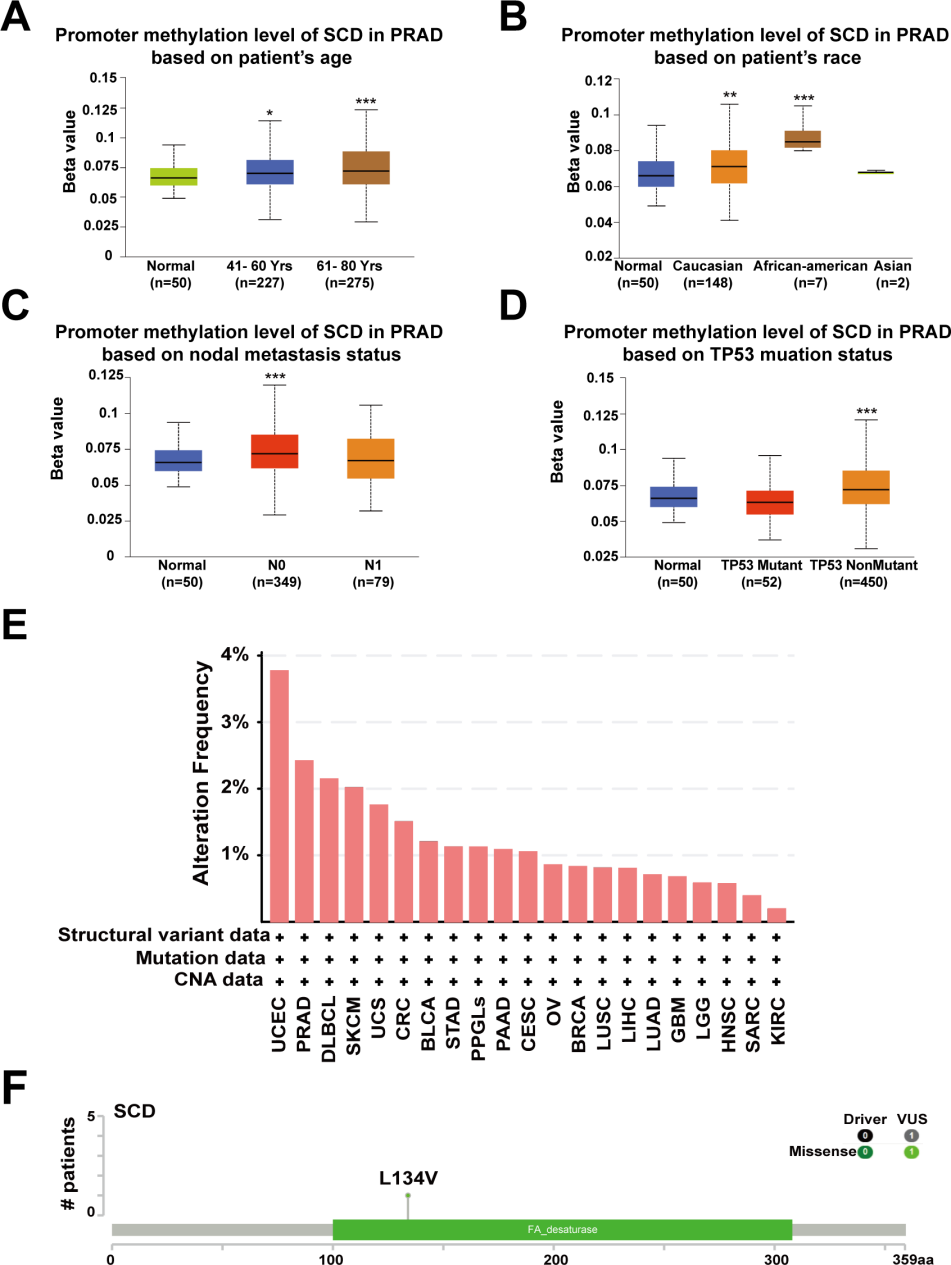
Promoter methylation and genetic alterations of the *SCD* gene. Box plots evaluating *SCD* promoter methylation levels in PRAD among different groups of patients based on clinical parameters including patients’ age **(A)**, race **(B)**, nodal metastasis status **(C)**, *TP53* mutation status **(D)** using the UALCAN database. Multiple groups were compared using one-way ANOVA followed by Bonferroni’s multiple comparisons for every two groups. **(E)** Alterations summary of *SCD* in different tumors. **(F)** The most common *SCD* genetic mutation in PRAD analyzed by the cBioPortal database. ^*^ *P <*0.05; ^**^ *P <*0.01; ^***^ *P <*0.001.

Abnormal DNA methylation is closely related to the occurrence and development of cancer. Therefore, we performed UALCAN analysis using TCGA-PRAD samples to compare the promoter methylation levels of *SCD*. The results suggested that promoter methylation levels of *SCD* were elevated in patients aged 41-80 years, especially 61-80 years (**Figure 3A**), in Caucasian and African-American races (**Figure 3B**), in those with N0 nodal metastasis status (**Figure 3C**) and in patients with non-mutant *TP53* (**Figure 3D**). Despite the traditional perception of DNA methylation as a transcriptional silencing mechanism, emerging research indicates that high promoter methylation levels in malignant tumors are associated with elevated gene expression (35, 36). The most frequently postulated mechanism posits that the hypermethylation of promoter DNA obstructs the binding of known inhibitory transcription factors, ultimately fostering active gene transcription (37).

Furthermore, we investigated the genetic alteration characteristics of *SCD* in the TCGA cohort through the cBioPortal network. According to the results, the frequency of *SCD* alteration in PRAD patients was 2.43% in 494 cases (**Figure 3E**). Particularly, mutations in the FA desaturase domain containing the *SCD* active site, with L134V (Leucine converts to Valine) being the most common locus, were observed in PRAD (**Figure 3F**). Both the original (Leu) and the substituted (Val) residues are non-polar with hydrophobic side chains and have only a slight difference. Therefore, we speculate that the L134V mutation may increase PRAD risk by enhancing SCD function. However, further clinical patient data is required to confirm this relationship. These findings revealed that the abnormal expression of *SCD* in specific cancers may stem from genetic alterations and promoter hypermethylation, providing a deeper understanding of PRAD tumorigenesis and guiding potential treatment strategy.

### 3.3 Immune cell infiltration and TME correlated with *SCD* expression

**Figure 4:**
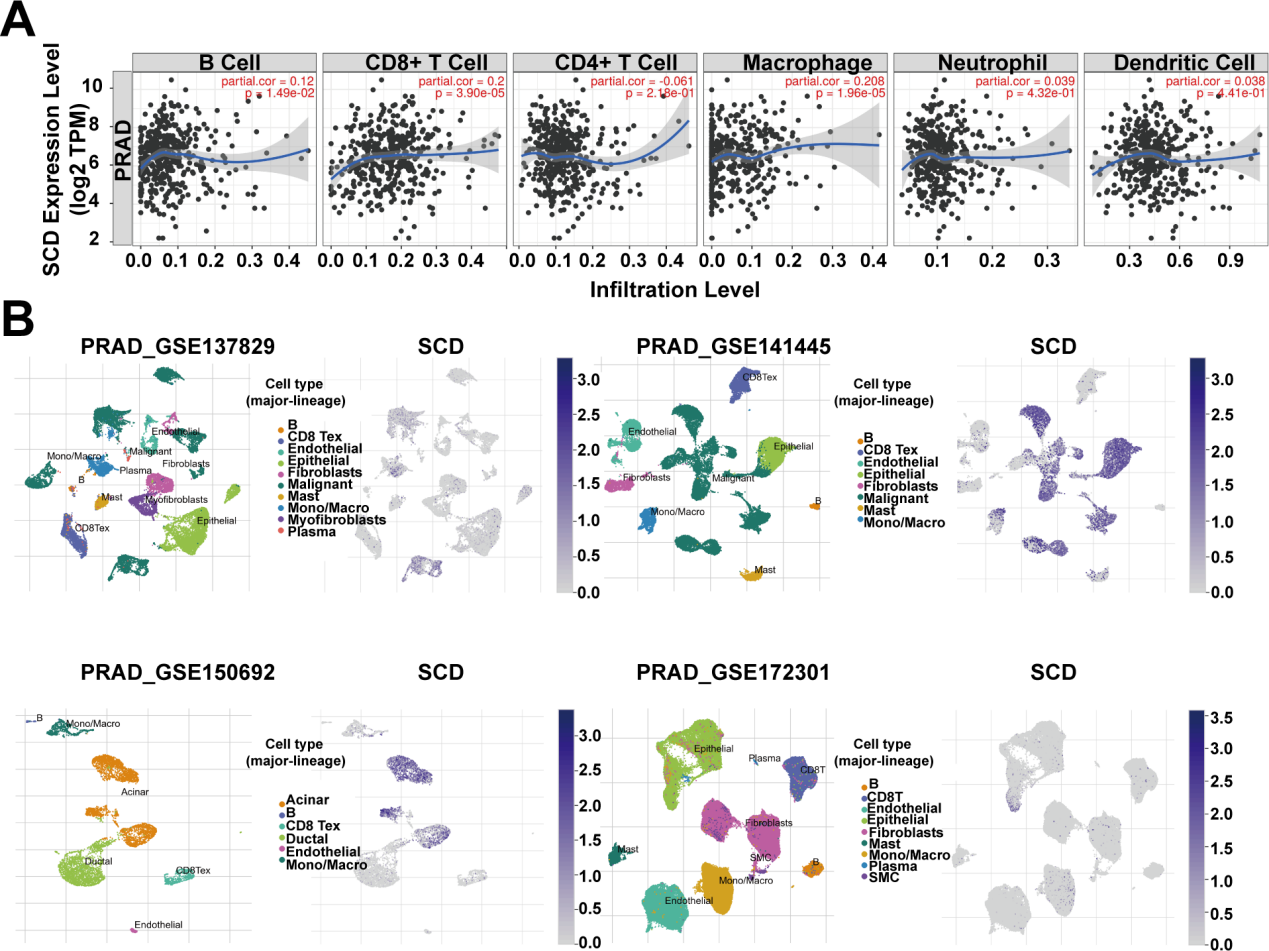
*SCD* expression in tumor microenvironment. **(A)** Relationship between the *SCD* expression and immune cell infiltration in PRAD using the TIMER tool. Spearman’s correlation was used to perform this association analysis. **(B)** *SCD* expression at single-cell resolution in different PRAD datasets from TISCH database.

The association between *SCD* expression and the infiltration of immune cells was evaluated across TCGA-PRAD using the TIMER tool. The correlation between *SCD* expression and markers of various immune cells, including B cells, T cells, monocytes, macrophages, neutrophils, natural killer cells, and dendritic cells, was analyzed (**Supplementary Table S2**). While some immune cells, such as CD8+ T cells and macrophages, were positively associated with *SCD* expression, a greater number of immune cells showed a negative correlation (**Figure 4A**; **Supplementary Table S2**). Additionally, the correlation between *SCD* expression and immune checkpoints was assessed (**Table 1**). Notably, the activity of immunosuppressive molecules like LAG3, LGALS9, PD-L1(PDCD1), and CTLA4 was negatively correlated with *SCD* expression, whereas immune-activating molecules such as TNFRSF18, TNFSF4, and CD47 were positively correlated. This suggests that the promotive effects of *SCD* expression on PRAD progression may lead to the activation of immune cells in the tumor microenvironment.

**Table 1:** Correlation analysis of stearoyl-CoA desaturase 1 with immune checkpoints in PRAD.

Immune checkpoints	None		Purity	
	Correlation	*P*	Correlation	*P*
CD27	-0.17077	0.000131	-0.16236	0.000888
CD274	0.137593	0.002088	0.101659	0.03798
CD28	0.003434	0.939062	-0.04531	0.356026
CD40	-0.16306	0.000263	-0.15793	0.001228
CD40LG	0.03964	0.377379	0.021128	0.667046
CD47	0.294619	1.98E-11	0.265151	4.36E-08
CD70	-0.05012	0.264226	-0.04394	0.370822
CD80	0.012534	0.780231	-0.01705	0.728511
CSF1	-0.05731	0.201594	-0.06424	0.190352
CTLA4	-0.18786	2.53E-05	-0.16443	0.000761
HAVCR2	-0.04707	0.294391	-0.06695	0.172303
HLA-DQA1	-0.03016	0.501777	-0.04757	0.332342
ICOS	-0.03144	0.483855	-0.06511	0.184516
ICOSLG	0.092303	0.039491	0.07786	0.112382
LAG3	-0.2786	2.50E-10	-0.24021	6.92E-07
LGALS9	-0.21223	1.87E-06	-0.20107	3.67E-05
PDCD1	-0.19844	8.14E-06	-0.17138	0.000447
PVR	0.342407	3.82E-15	0.325377	9.72E-12
SIRPA	0.015657	0.72744	0.004948	0.919756
TIGIT	-0.04081	0.363457	-0.05128	0.296127
TNFRSF18	-0.25476	8.10E-09	-0.20142	3.42E-05
TNFRSF4	-0.2783	3.12E-10	-0.24958	2.66E-07
TNFSF18	0.127304	0.004436	0.135159	0.005702
TNFSF4	0.201286	6.26E-06	0.17079	0.000468

In addition, four datasets (PRAD_GSE137829, PRAD_GSE141445, PRAD_GSE150692, and PRAD_GSE172301) from the TISCH database were utilized to explore *SCD* expression in the TME. The results revealed that malignant cells, mono/macro cells, epithelial cells, and acinar cells exhibited higher levels of *SCD* expression compared to other cell types (**Figure 4B**). These findings suggest that *SCD* is highly expressed not only in malignant cells but also in immune and stromal cells. In summary, *SCD* shows a broad expression pattern across various cell types within the TME, highlighting its significant immunomodulatory potential.

### 3.4 Gene association network analysis unveiling potential SCD roles in PRAD

**Figure 5:**
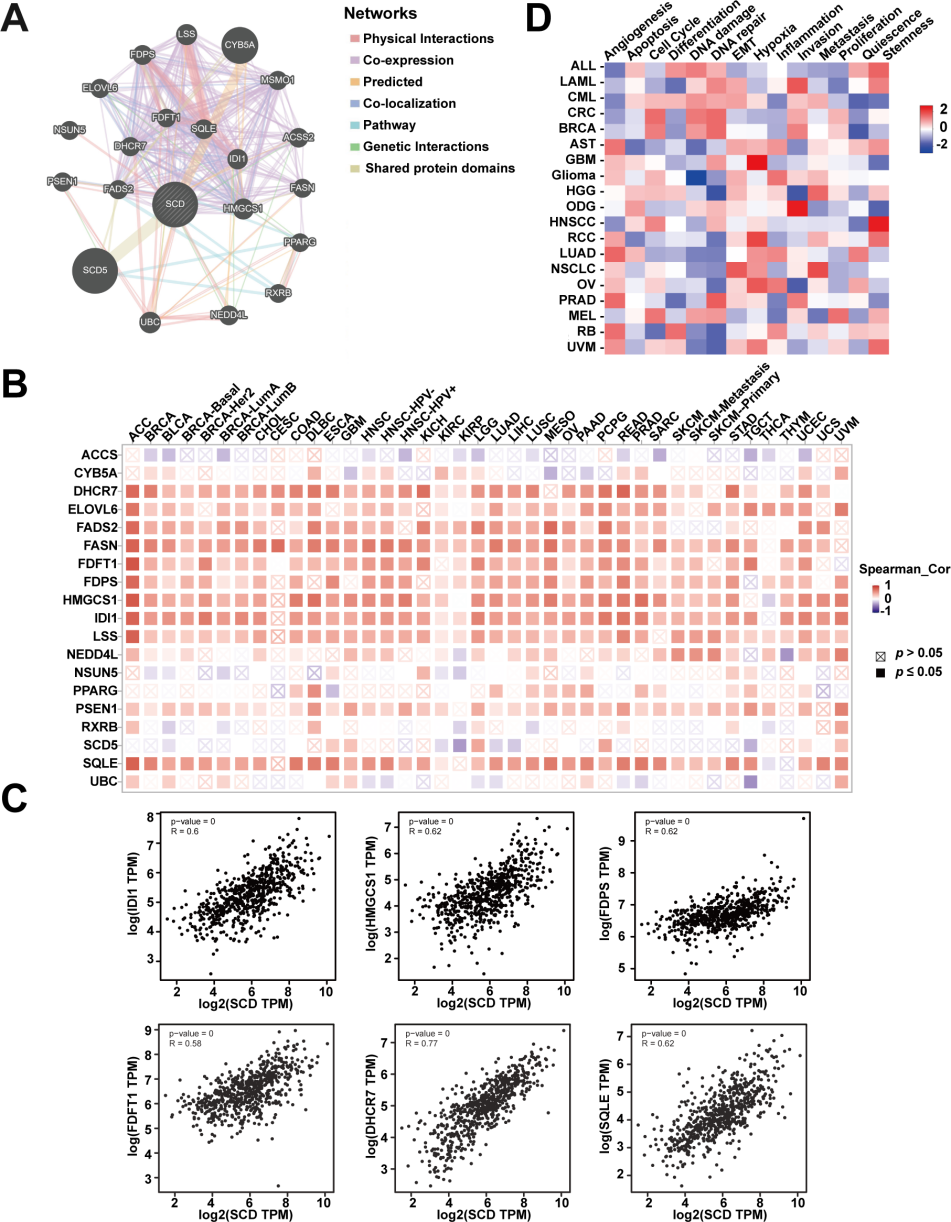
Analyses of *SCD*-related genes. **(A)** *SCD*-related gene network analyzed in the GeneMANIA database. **(B)** The expression of *SCD-*related genes in different cancers. **(C)** Scatter plots of correlation analyses of the expression of *SCD* and *SCD*-related genes. Spearman’s correlation was used to perform this association analysis. **(D)** Correlations between SCD and 14 functional states in different cancers.

In order to explore the biological functions of SCD, gene association analysis was conducted with the GeneMANIA and 20 *SCD*-related genes were identified (**Figure 5A**). The related genes were mainly involved in regulating sterol biosynthesis, steroid biosynthesis, and lipid biosynthesis processes according to physical interactions, co-expression, prediction and co-localization (**Supplementary Table S3**). Similar correlations were shown in **Figure 5B** from TIMER database at the pan-cancer level. The top six genes in the relevance ranking were identified, including isopentenyl-diphosphate delta-isomerase 1 (IDI1), 3-hydroxy-3-methylglutaryl-coenzyme A synthase 1 (HMGCS1), farnesyl diphosphate synthase (FDPS), farnesyl-diphosphate farnesyltransferase 1 (FDFT1), 7-dehydrocholesterol reductase (DHCR7), squalene epoxidase (SQLE). Scatter plots showing the expression of these genes, along with *SCD*, are presented in **Figure 5C**.

In addition, single-cell transcriptome sequencing is a key technique for analyzing the potential functions of candidate molecules at the single-cell level. Using the CancerSEA, we investigated the potential functions of SCD at single cell levels in various cancers. The results highlighted a noteworthy correlation between *SCD* expression and inflammation in pan-cancer (**Figure 5D**). Moreover, *SCD* was also positively correlated with epithelial-mesenchymal transition (EMT) and hypoxia, suggesting that *SCD* is a key factor in promoting cancer malignancy.

### 3.5 Functional enrichment analysis of SCD relationships with cancer cell fate

**Figure 6:**
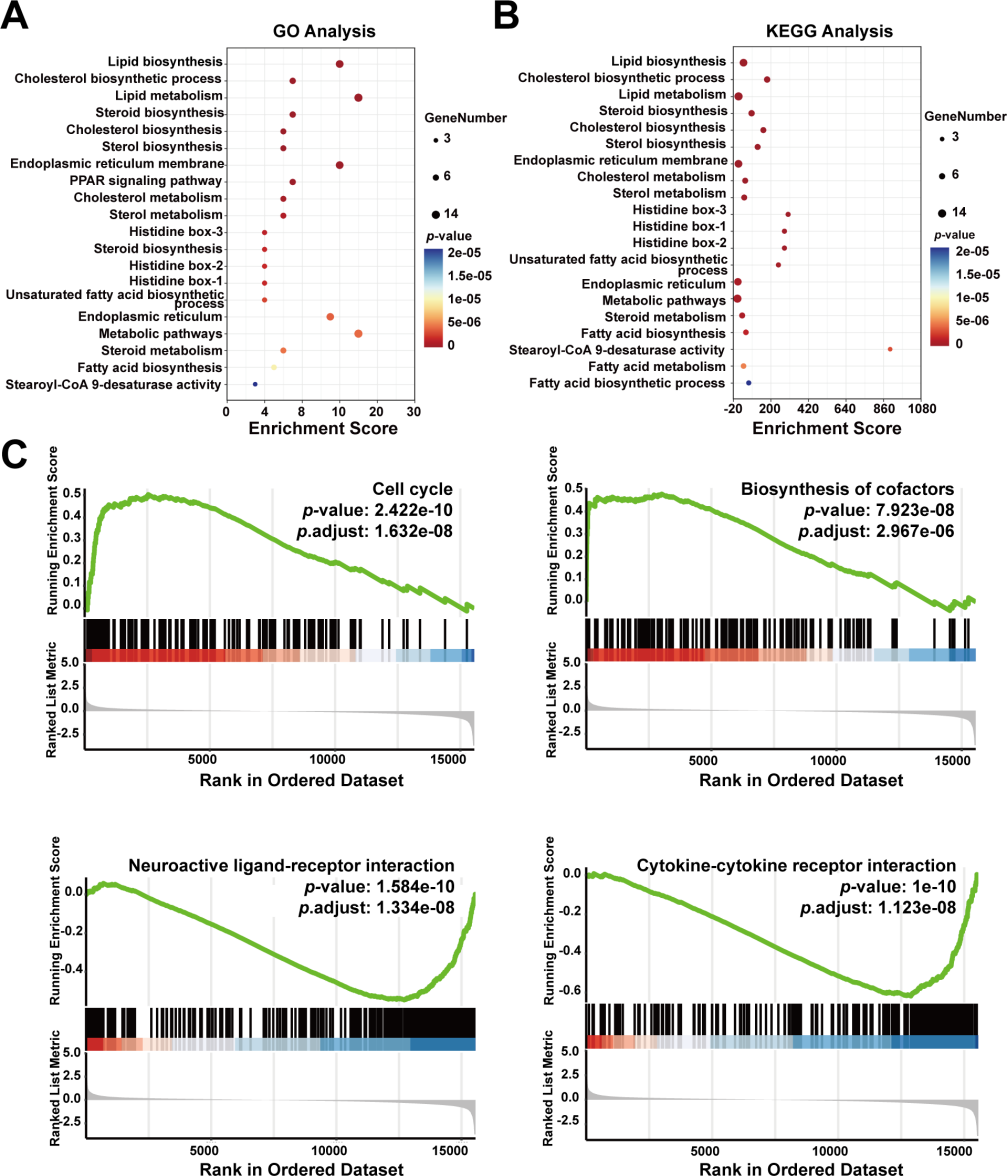
Enrichment analysis of *SCD*-related genes in cancer functional states. **(A)** GO enrichment analysis of *SCD*-related genes. **(B)** KEGG enrichment analysis of *SCD*-related genes. The size of the bubble represented the number of enriched genes, and the color correlated with the *p*-value. **(C)** Functional enrichment analyses of *SCD* in PRAD by GSEA.

To further explore the functions of SCD based on the 20 identified SCD-related genes, we performed GO and KEGG analyses, which are essential tools in functional genomics. GO focuses on classifying and describing gene functions across three main categories—molecular function, biological process, and cellular component. KEGG analysis is primarily used to explore how genes interact within biological pathways, such as those involved in metabolic processes, signal transduction, and disease mechanisms. Our GO analysis revealed that SCD was mainly enriched in biological processes, including lipid biosynthesis and metabolism, cholesterol biosynthesis, steroid biosynthesis, and endoplasmic reticulum membrane functions (**Figure 6A**). Our KEGG analysis revealed major enrichment in metabolic processes, including lipid biosynthesis, cholesterol biosynthesis, endoplasmic reticulum membrane functions (**Figure 6B**).

To explore SCD roles in cellular events, the GSEA tool was used, which revealed a positive correlation between high *SCD* expression and the cell cycle (*P* = 1.632e-08) as well as biosynthesis of cofactors (*P* = 2.967e-06) (**Figure 6C**). Conversely, *SCD* expression was negatively correlated with neuroactive ligand-receptor interaction (*P* = 1.334e-08) and cytokine-cytokine receptor interaction (*P* = 1.123e-08) (**Figure 6C**). This suggested potential alterations in the immune microenvironment that may facilitate immune escape and drug resistance.

### 3.6 SCD regulation of PRAD progression in functional assays

**Figure 7:**
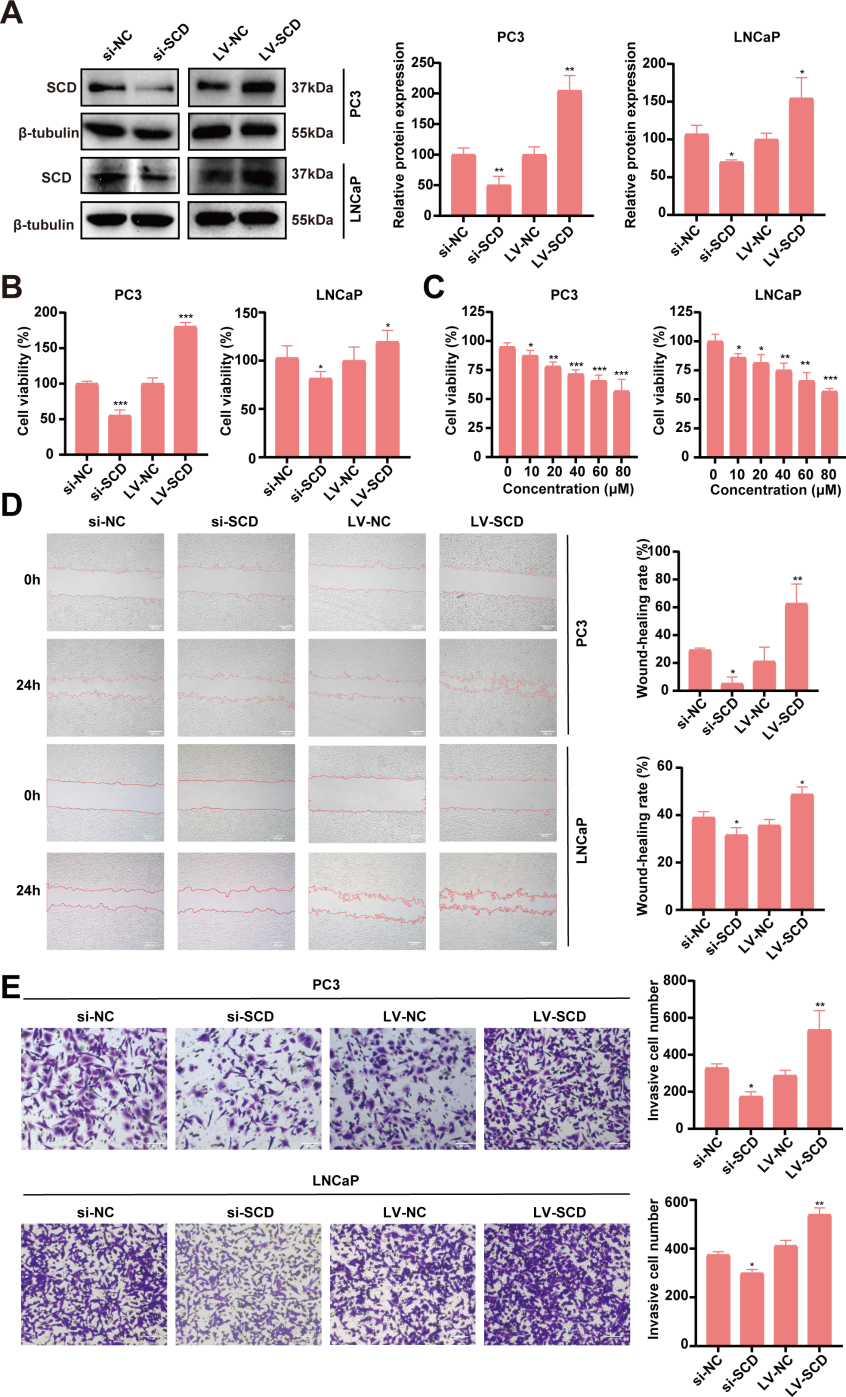
Validation of SCD roles in PRAD *in vitro*. **(A)** *SCD* knockdown or overexpression in PC3 cells or LNCaP cells was conducted with siRNA (si) or *SCD*-loaded lentivirus (LV-SCD). SCD expression was analyzed by Western blot, with β-tubulin as a control. Quantification of protein levels were shown in the right. **(B)** The viability of PRAD cells was affected by *SCD* knockdown or overexpression in MTT assays. **(C)** SCD inhibitor, MF438, dose-dependently suppressed the viability of PRAD cells. SCD expression levels affected the migration **(D)** and the invasion **(E)** of PRAD cells. Representative images from three independent experiments are shown. Quantification of migration and invasion were conducted with the ImageJ software. Data are presented as the mean ± SD (n = 3). Student’s *t*-test was used to analyze the significance of the differences. ^*^ *P <*0.05; ^**^ *P <*0.01; ^***^ *P <*0.001.

The bioinformatic analyses suggested SCD plays important roles in the pathogenesis and progression of PRAD. To validate SCD roles directly, functional studies with SCD overexpression and knockdown were conducted in PRAD cell lines. Two PRAD cell lines, PC3 and LNCaP, were treated with siRNA and/or lentivirus to knock down or overexpress SCD (**Figure 7A**). In MTT assays, siRNA knockdown of SCD led to a significant decrease in cell viability, while its overexpression through lentivirus transfection resulted in increased viability in both of the cell lines (**Figure 7B**). Additionally, treatment of both cell lines with the SCD inhibitor MF438 resulted in a concentration-dependent inhibition of cell viability (**Figure 7C**). These findings demonstrate the pivotal role of SCD protein in promoting PRAD cell proliferation.

The impact of SCD on PRAD cell migration and invasion was further investigated using PC3 and LNCaP cell lines. In the scratch assays for analyzing cell migration, SCD knockdown significantly reduced the migration capacity of both PRAD cell lines compared to the negative control (si-NC) group, as shown in **Figure 7D**. In contrast, overexpression of SCD substantially enhanced migration ability relative to the control group (**Figure 7D**). Similarly, in the transwell invasion assays, SCD knockdown markedly decreased the invasion ability of both PRAD cell lines compared to the si-NC group (**Figure 7E**). Conversely, overexpression of SCD significantly boosted invasion capability compared to the control group (**Figure 7E**). These findings collectively suggest that SCD plays a crucial role in regulating PRAD aggressiveness, underscoring its potential as a therapeutic target for PRAD treatment.

## 4 Discussion

In this study, the impact of *SCD* gene expression and the functional roles of the SCD protein in prostate adenocarcinoma (PRAD) were comprehensively analyzed using bioinformatic tools and functional experiments. Our findings revealed that the *SCD* gene is highly expressed in PRAD patients, and the SCD protein promotes the proliferation, invasion, and migration of PRAD tumor cells. Our research aligns with other studies that have identified a significant correlation between high *SCD* expression and an unfavorable prognosis in various tumor types (38–40). SCD has been extensively studied for its role in promoting tumor initiation, progression, metastasis, and stemness (41). Collectively, these findings suggest that SCD could serve as both a biomarker and a therapeutic target for PRAD patients.

We have revealed that *SCD* is notably overexpressed in Caucasian patients, as well as in patients with Gleason score 8, stage N1 lymph node metastasis, or ERG fusion. Racial disparities in prostate cancer incidence are largely rooted in inherent genetic factors that are non-modifiable, underscoring the necessity of targeted screening in specific populations for early detection (42). Early detection facilitates prompt treatment, which can begin even during adolescence, and is crucial for improving outcomes (43, 44). The Gleason scoring system is a widely accepted method for evaluating the malignancy of prostate cancer, based on the differentiation degree of cancer cells and pathophysiological changes in prostate tissues. A score of 8 indicates poor differentiation and categorizes the cancer as high-risk (45). Lymph node metastasis, indicated by stage N1, signifies the progression of cancer cells through lymphatic vessels, leading to tumor spread and often necessitating immediate intervention through surgery, chemotherapy, radiotherapy, or targeted therapy to manage symptoms such as swelling, skin depression, and local redness (46). The most common molecular alteration in prostate cancer is the fusion of ERG and TMPRSS2, a widespread event associated with poor prognosis (47, 48). In summary, effective management of prostate cancer requires a holistic approach that considers individual patient characteristics and disease progression, with early detection, accurate diagnosis, and personalized treatment plans being essential for enhancing prognosis.

Interestingly, the elevated *SCD* expression in PRAD was accompanied by promoter hypermethylation and genetic mutations. Although DNA methylation represses gene transcription in many other circumstances, promoter hypermethylation is usually associated with enhanced gene expression in malignant tumors (35, 36, 49). This is explained by that promoter hypermethylation obstructs the binding of known inhibitory transcription factors, ultimately fostering gene transcription (37). Gene mutations and methylation play crucial roles in tumor development (50, 51). Specifically, gene mutations alter the function of critical genes, driving tumor progression. Meanwhile, methylation regulates gene expression and cellular function, thereby influencing tumor development. These two mechanisms are intricately intertwined and work synergistically to promote tumor formation and advancement. An in-depth study of these two mechanisms is crucial for the advancement of novel tumor diagnostic methodologies and therapeutic strategies (51). Among various cancer types, the mutation frequency of *SCD* in prostate cancer patients stands at 2.43%, making it the second highest. This provides a clue for us to find *SCD* mutations, that is, L134V was observed as the most common locus in PRAD. It has been documented that the substitution of leucine (Leu) for valine (Val) within the amino acid sequence of a protein leads to significant alterations in its three-dimensional structure. These modifications can have profound effects on the protein’s activity, stability, and its ability to interact with other molecules, ultimately establishing a correlation with a wide spectrum of diseases (52, 53). It’s reported that mutations in the type II steroid 5 alpha-reductase (SRD5A2) gene, which replaces valine with leucine at codon 89, reduced the risk of prostate cancer in Italian patients (54).

This study showed that *SCD* expression exhibits a negative correlation with the expression of various immune cell markers, suggesting a reduction in immune cell infiltration in PRAD. This reduction likely contributes to a tumor-friendly immune microenvironment, facilitating PRAD progression. However, *SCD* expression was positively associated with the infiltration of CD8+ T cells and macrophages in the TCGA-PRAD datasets. CD8+ T cells a are known to enhance the clearance of tumor cells, and M1 macrophages secrete pro-inflammatory factors and cytotoxic molecules, effectively targeting and eliminating tumor cells. Moreover, *SCD* expression was negatively correlated with immune checkpoints such as PD-L1 and CTLA4. Therefore, it should not be concluded that *SCD* expression enhances the anti-tumor effects of immune cells. Instead, the enhancement of certain immune responses may be accompanied by PRAD progression, which is the consequence of *SCD* expression. Currently, numerous emerging technologies and materials have shown promising outcomes by specifically targeting the TME in immunotherapy. These include biological materials such as lipid nanoparticles, organic biomaterials, and metal oxide nanomaterials, which can reprogram the TME, thereby enhancing the efficacy of immunotherapy and strengthening the anti-tumor effect (55, 56). Additionally, chimeric antigen receptor (CAR) T cell therapy, a novel precision-targeted approach, has demonstrated remarkable success in treating hematological malignancies (57). Immune checkpoint inhibitors (ICI), another crucial therapeutic modality, have the potential to cure cancer by modulating the immune system and reducing drug resistance (58). Given these advancements, SCD presents considerable therapeutic potential within the realm of immunotherapy. Its application could be further explored and optimized through combination therapies or the innovative use of biological materials in future research and development.

Lipid metabolism plays a critical role in tumor development. Several drugs targeting lipid metabolism have been developed for the treatment of prostate cancer, including statins, proprotein convertase subtilisin/kexin type 9 (PCSK9) inhibitors, and sterol regulatory element-binding protein (SREBP) inhibitors (59–61). Lipids not only serves as a source of energy but also maintains redox homeostasis for cancer cells. Cancer cells have a tendency to synthesize lipids *de novo* and the desaturation process of fatty acids is of particular significance in lipid synthesis. Our study has revealed a strong correlation between the expression of *SCD* and the expression of IDI1, HMGCS1, FDPS, FDFT1, DHCR7, and SQLE genes in the pathogenesis of PRAD. These genes play a pivotal role in the biosynthesis of bioactive substances, notably cholesterol, and are extensively implicated in tumor lipid metabolism. They are consistently overexpressed in a wide range of cancers, indicating a potential synergistic contribution to the promotion of tumor initiation and development, particularly in malignancies involving cholesterol metabolism (62–69). The specific mechanism by which targeting lipid desaturation may combat cancer includes reducing membrane fluidity to inhibit metastasis, increasing the lipid toxicity of saturated fatty acids, and decreasing the polarization of tumor stem cells and the secretion of factors such as interleukin-6 and nitric oxide by mesenchymal stem cells (70–72). Further research is required to fully elucidate the roles of these lipid metabolism-associated genes in cancer pathogenesis and to explore their potential as therapeutic targets.

The enrichment analyses further investigate the biological functions and pathways that *SCD* may be implicated in PRAD. Interestingly, we observed a negative correlation between *SCD* expression and cytokine-cytokine receptor interaction, a process closely linked to immune activity. Signaling between cytokines and cytokine receptors is essential for the production, survival, and homeostasis of immune cells, and for generating immune responses in response to external stimuli (73). Cytokines play a pivotal role in the pathogenesis and treatment of cancer (74). A reduction in cytokine-cytokine receptor interaction may alter the TME, potentially enabling tumors to evade immune surveillance and develop treatment resistance. Combined with bioinformatic analyses, our functional studies clearly indicate that targeting SCD is a promising strategy for combating PRAD, as has been extensively reported in cases of other cancers (75–77). For example, T-3764518, a novel oral small molecule inhibitor of SCD, has demonstrated efficacy in inhibiting the growth of HCT-116 cell xenografts in mouse models of colorectal cancer (78). The combination of SCD inhibitor A939572 with Tisirolimus synergistically inhibits the growth of clear cell renal cell carcinoma both *in vitro* and *in vivo* (79). In addition, SCD has potential as a prognostic marker in various cancers and may aid in overcoming drug resistance (80, 81). Therefore, SCD emerges as a potential target for cancer therapy, exhibiting robust anti-cancer effects in both *in vivo* and *in vitro* models, and showing promise in the realm of overcoming drug resistance.

In conclusion, this study systematically explored the roles of SCD in PRAD through bioinformatic and experimental analyses. The gene and protein expression of SCD were significantly upregulated in PRAD. Importantly, this upregulation was associated with specific clinicopathological features and the TME, highlighting its potential as a biomarker and therapeutic target for PRAD. These findings are significant for understanding PRAD pathogenesis and provide insights for targeted interventions in this aggressive cancer.

## Data Availability

The original contributions presented in the study are included in the article/**Supplementary Material**. Further inquiries can be directed to the corresponding author.
