# Supplementary material for: Comprehensive analysis of stearoyl-coenzyme A desaturase in prostate adenocarcinoma: insights into gene expression, immune microenvironment and tumor progression
Source: Front Immunol. 2024 Sep 16;15:1460915. doi: 10.3389/fimmu.2024.1460915 (PMC11439642; doi:10.3389/fimmu.2024.1460915)
Supplement: Supplementary file 1 [file DataSheet1.docx]

**Supplementary Material**

**Comprehensive analysis of Stearoyl-coenzyme A desaturase in prostate adenocarcinoma: Insights into gene expression, immune microenvironment and tumor progression**

**Jie Wang^1†^, Liang Ying^1†^,** **He Xiong^1^, Duan-Rui Zhou^1^, Yi-Xuan Wang^1^,** **Hui-Lian Che^1^,** **Zhang-Feng Zhong^2^,** **Guo-Sheng Wu^1^** **and Yun-Jun Ge^1*^**

**1 Supplementary Tables**

**1.1 Supplementary Table S1.** Abbreviations of the cancers mentioned in this study.

| Abbreviations | Definition |
| --- | --- |
| AST | Astrocytoma |
| BLCA | Bladder urothelial carcinoma |
| BRCA | Breast invasive carcinoma |
| CESC | Cervical squamous cell carcinoma and endocervical adenocarcinoma |
| CHOL | Cholangiocarcinoma |
| CML | Chronic myelogenous leukemia |
| COAD | Colon adenocarcinoma |
| CRC | Colorectal cancer |
| DLBCL | Diffuse large B cell lymphoma |
| ESCA | Esophageal carcinoma |
| GBM | Glioblastoma multiforme |
| GBMLGG | Glioma |
| HGG | High-Grade Glioma |
| HNSC/HNSCC | Head and Neck squamous cell carcinoma |
| KICH | Kidney Chromophobe |
| KIRC | Kidney renal clear cell carcinoma |
| KIRP | Kidney renal papillary cell carcinoma |
| LAML | Acute Myeloid Leukemia |
| LGG | Brain Lower Grade Glioma |
| LIHC | Liver hepatocellular carcinoma |
| LUAD | Lung adenocarcinoma |
| LUSC | Lung squamous cell carcinoma |
| MEL | Melanoma |
| NSCLC | Non-small cell lung cancer |
| ODG | Oligodendroglioma |
| OV | Ovarian serous cystadenocarcinoma |
| PAAD | Pancreatic adenocarcinoma |
| PPGLs | Pheochromocytoma and paraganglioma |
| PRAD | Prostate adenocarcinoma |
| RB | Retinoblastoma |
| RCC | Renal cell carcinoma |
| READ | Rectum adenocarcinoma Esophageal carcinoma |
| SARC | Sarcoma |
| SKCM | Skin Cutaneous Melanoma |
| STAD | Stomach adenocarcinoma |
| THCA | Thyroid carcinoma |
| UCEC | Uterine Corpus Endometrial Carcinoma |
| UCS | Uterine Carcinosarcomas |
| UVM | Uveal Melanoma |

**1.2 Supplementary Table S2.** Correlations analysis of stearoyl-CoA desaturase 1 with immune cell markers in PRAD.

| Description | Gene markers | None | | Purity | |
| --- | --- | --- | --- | --- | --- |
|  |  | Correlation | *P* | Correlation | *P* |
| CD8^+^ T cell | CD8A | -0.09758 | 0.046468 | -0.46625 | 6.78E-24 |
|  | CD8B | -0.07072 | 0.149374 | -0.28527 | 3E-09 |
| T cell (general) | CD3D | -0.1805 | 0.000211 | -0.44041 | 3.27E-21 |
|  | CD3E | -0.1315 | 0.007204 | -0.47159 | 1.77E-24 |
|  | CD2 | -0.10334 | 0.034936 | -0.41958 | 3.27E-19 |
| B cell | CD19 | -0.1228 | 0.012089 | -0.30091 | 3.56E-10 |
|  | CD79A | -0.08867 | 0.070469 | -0.29489 | 8.21E-10 |
| Monocyte | CD86 | -0.07101 | 0.147728 | -0.37444 | 2.51E-15 |
|  | CSF1R | -0.08002 | 0.102716 | -0.44448 | 1.28E-21 |
| TAM | CCL2 | -0.20001 | 4.03E-05 | -0.39651 | 3.75E-17 |
|  | CD68 | -0.02235 | 0.648916 | -0.31139 | 7.92E-11 |
|  | IL10 | -0.04728 | 0.335436 | -0.30276 | 2.74E-10 |
| M1 Macrophage | NOS2 | -0.05937 | 0.226366 | -0.28533 | 2.98E-09 |
|  | IRF5 | 0.118661 | 0.015376 | -0.12548 | 0.010325 |
|  | PTGS2 | 0.028056 | 0.567788 | -0.32561 | 9.37E-12 |
| M2 Macrophage | CD163 | -0.00719 | 0.883503 | -0.27945 | 6.42E-09 |
|  | VSIG4 | -0.02571 | 0.60044 | -0.33476 | 2.23E-12 |
|  | MS4A4A | -0.07884 | 0.107881 | -0.31817 | 2.91E-11 |
| Neutrophils | CEACAM8 | 0.070912 | 0.148308 | -0.05944 | 0.225804 |
|  | ITGAM | -0.06012 | 0.220411 | -0.39115 | 1.07E-16 |
|  | CCR7 | -0.08485 | 0.083517 | -0.43215 | 2.11E-20 |
| Natural killer cell | KIR2DL1 | 0.035695 | 0.467251 | -0.11802 | 0.015903 |
|  | KIR2DL3 | -0.02951 | 0.547909 | -0.02404 | 0.624489 |
|  | KIR2DL4 | 0.025363 | 0.605536 | -0.17264 | 0.000398 |
|  | KIR3DL1 | 0.046659 | 0.341883 | -0.15993 | 0.001049 |
|  | KIR3DL2 | -0.01969 | 0.688452 | -0.08447 | 0.08491 |
|  | KIR3DL3 | 0.001932 | 0.968616 | 0.096849 | 0.048107 |
|  | KIR2DS4 | -0.09212 | 0.060166 | -0.10983 | 0.0249 |
| Dendritic cell | HLA-DPB1 | -0.18041 | 0.000218 | -0.46257 | 1.69E-23 |
|  | HLA-DQB1 | -0.12477 | 0.010806 | -0.28777 | 2.15E-09 |
|  | HLA-DRA | -0.05062 | 0.302462 | -0.43951 | 4.00E-21 |
|  | HLA-DPA1 | -0.0749 | 0.126735 | -0.43563 | 9.67E-21 |
|  | CD1C | -0.09123 | 0.06271 | -0.47163 | 1.75E-24 |
| Th1 | NRP1 | 0.114206 | 0.019702 | -0.03078 | 0.530835 |
|  | ITGAX | -0.08437 | 0.085272 | -0.31723 | 3.34E-11 |
|  | TBX21 | -0.1301 | 0.007814 | -0.39129 | 1.05E-16 |
|  | STAT1 | 0.265446 | 3.73E-08 | -0.17905 | 0.000238 |
|  | IFNG | -0.08183 | 0.09515 | -0.20022 | 3.82E-05 |
|  | TNF | -0.09022 | 0.065699 | -0.33617 | 1.78E-12 |
| Th2 | GATA3 | -0.08494 | 0.083212 | -0.53176 | 8.19E-32 |
|  | STAT6 | 0.096534 | 0.048872 | -0.27637 | 9.54E-09 |
|  | STAT5A | -0.08868 | 0.070463 | -0.49027 | 1.33E-26 |
|  | IL13 | -0.14568 | 0.002865 | -0.09029 | 0.065488 |
| Tfh | BCL6 | -0.00527 | 0.914465 | -0.32228 | 1.56E-11 |
|  | IL21 | 0.047553 | 0.332703 | -0.09494 | 0.052698 |
| Th17 | STAT3 | 0.31755 | 3.19E-11 | -0.24213 | 5.61E-07 |
|  | IL17A | 0.010387 | 0.832509 | -0.25626 | 1.12E-07 |
| Treg | FOXP3 | 0.065012 | 0.185174 | -0.23584 | 1.11E-06 |
|  | CCR8 | 0.07577 | 0.122384 | -0.19312 | 7.21E-05 |
|  | STAT5B | 0.110141 | 0.024541 | -0.30078 | 3.63E-10 |
|  | TGFB1 | -0.12851 | 0.008606 | -0.40997 | 2.47E-18 |
| Exhausted T cell | PDCD1 | -0.17138 | 0.000447 | -0.36487 | 1.41E-14 |
|  | CTLA4 | -0.16443 | 0.000761 | -0.31385 | 5.52E-11 |
|  | LAG3 | -0.24021 | 6.92E-07 | -0.37772 | 1.37E-15 |
|  | HAVCR2 | -0.06695 | 0.172303 | -0.35838 | 4.40E-14 |
|  | GZMB | -0.05982 | 0.222878 | -0.36702 | 9.63E-15 |
|  |  |  |  |  |  |
| TAM, Tumor-associated macrophage; Th1, T helper cell 1; Th2, T helper cell 2; Tfh, follicular helper T cell; Th17, T helper cell 17;Treg, regulatory T cells. | | | | | |

**1.3 Supplementary Table S3** SCD-related functional states in PRAD from GeneMANIA database.

| Function | FDR | Genes in network | Genes in genome |
| --- | --- | --- | --- |
| Secondary alcohol biosynthetic process | 3.97E-19 | 11 | 62 |
| Sterol biosynthetic process | 3.97E-19 | 11 | 62 |
| Regulation of sterol biosynthetic process | 1.38E-18 | 10 | 42 |
| Regulation of cholesterol metabolic process | 1.40E-18 | 10 | 44 |
| Cholesterol biosynthetic process | 1.40E-18 | 10 | 44 |
| Cholesterol metabolic process | 2.34E-18 | 11 | 79 |
| Regulation of alcohol biosynthetic process | 7.13E-17 | 10 | 65 |
| Sterol metabolic process | 8.15E-17 | 11 | 110 |
| Secondary alcohol metabolic process | 1.21E-16 | 11 | 115 |
| Regulation of steroid biosynthetic process | 2.30E-16 | 10 | 75 |
| Alcohol biosynthetic process | 4.00E-16 | 11 | 130 |
| Regulation of steroid metabolic process | 2.95E-15 | 10 | 98 |
| Steroid biosynthetic process | 2.95E-15 | 11 | 157 |
| Slcohol metabolic process | 2.35E-14 | 12 | 284 |
| Organic hydroxy compound biosynthetic process | 4.24E-14 | 11 | 202 |
| Steroid metabolic process | 3.12E-13 | 11 | 243 |
| Regulation of lipid biosynthetic process | 3.33E-13 | 10 | 159 |
| Regulation of lipid metabolic process | 7.26E-13 | 11 | 265 |
| Acyl-CoA metabolic process | 1.22E-05 | 5 | 73 |
| Thioester metabolic process | 4.19E-05 | 5 | 94 |
| Fatty-acyl-CoA metabolic process | 8.83E-05 | 4 | 41 |
| Thioester biosynthetic process | 0.000146 | 4 | 48 |
| Ribonucleoside bisphosphate metabolic process | 0.000146 | 5 | 127 |
| Acyl-CoA biosynthetic process | 0.000146 | 4 | 48 |
| Nucleoside bisphosphate metabolic process | 0.000146 | 5 | 127 |
| Purine nucleoside bisphosphate metabolic process | 0.000146 | 5 | 127 |
| Oxidoreductase activity, acting on paired donors, with Incorporation or reduction of molecular oxygen | 0.000272 | 5 | 145 |
| Purine nucleoside bisphosphate biosynthetic process | 0.000336 | 4 | 62 |
| Nucleoside bisphosphate biosynthetic process | 0.000336 | 4 | 62 |
| Ribonucleoside bisphosphate biosynthetic process | 0.000336 | 4 | 62 |
| Isoprenoid biosynthetic process | 0.000447 | 3 | 17 |
| Fatty acid derivative biosynthetic process | 0.001682 | 4 | 94 |
| Isoprenoid metabolic process | 0.004037 | 4 | 118 |
| Fatty acid metabolic process | 0.007045 | 5 | 295 |
| Purine ribonucleotide biosynthetic process | 0.007703 | 4 | 141 |
| Purine nucleotide biosynthetic process | 0.00882 | 4 | 148 |
| Ribonucleotide biosynthetic process | 0.00882 | 4 | 148 |
| Ribose phosphate biosynthetic process | 0.009788 | 4 | 153 |
| Outer membrane | 0.011084 | 4 | 161 |
| Organelle outer membrane | 0.011084 | 4 | 161 |
| Fatty acid derivative metabolic process | 0.011084 | 4 | 159 |
| Purine-containing compound biosynthetic process | 0.012786 | 4 | 168 |
| Sulfur compound biosynthetic process | 0.013385 | 4 | 171 |
| Nucleotide biosynthetic process | 0.024588 | 4 | 201 |
| Nucleoside phosphate biosynthetic process | 0.029035 | 4 | 211 |
| Prenyltransferase activity | 0.035893 | 2 | 13 |
| Unsaturated fatty acid metabolic process | 0.07018 | 3 | 102 |
| Terpenoid metabolic process | 0.070731 | 3 | 103 |
| Fatty acid biosynthetic process | 0.079711 | 3 | 108 |
| FDR, false discovery rate. |  |  |  |

1. **Supplementary Figures**

**2.1 Supplementary Figure S1**


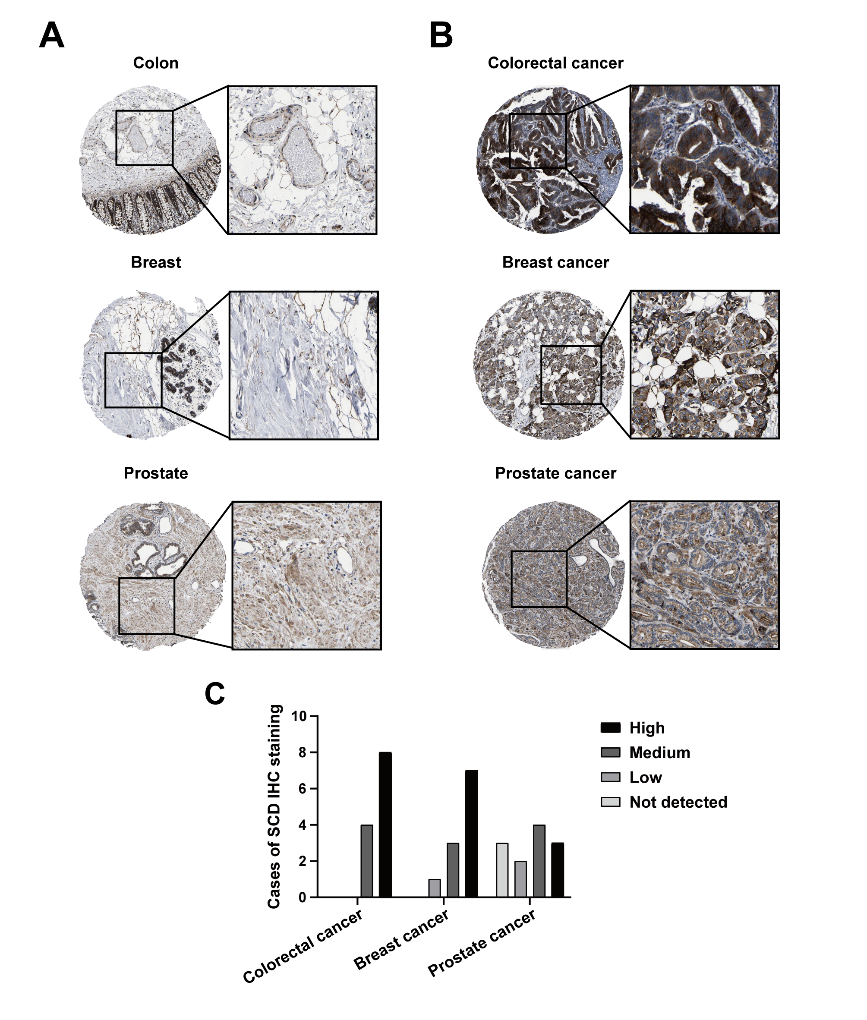


**Figure S1. Representative immunohistochemical images of SCD protein expression in normal tissues and the corresponding cancers from HPA database.** (A) SCD expression in normal colon, breast and prostate tissues. (B) SCD expression in colorectal cancer, breast cancer and PRAD tissues. (C) The quantification of SCD IHC staining intensities in the cases of high, medium, low and not detected.

- 1. **Supplementary Figure S2**


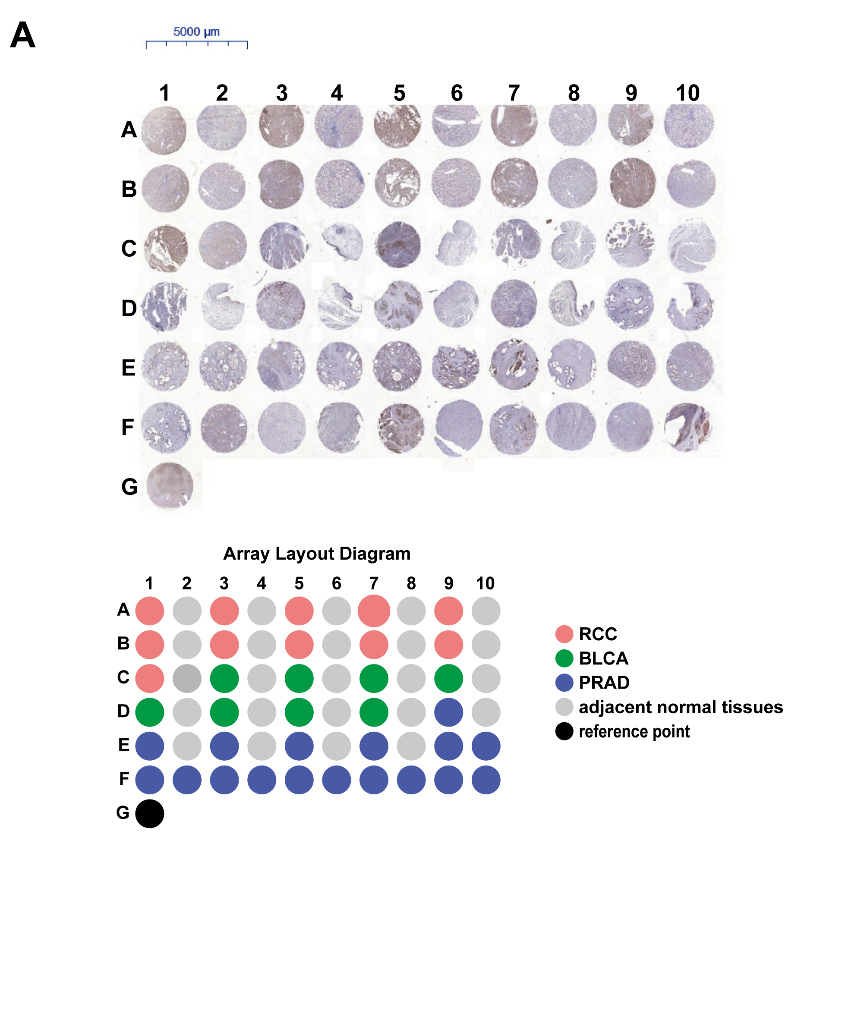


**Figure S2. Immunohistochemical analysis of SCD protein expression in normal tissues and the corresponding cancers in a tissue microarray.** The upper panel displays the IHC results, while the lower panel illustrates the arrangement of tumor tissues in the microarray, highlighted in different colors. Scale bar: 5000 μm.
